# Inflammatory Back Pain and Psoriasis: Expecting Spondyloarthritis, Discovering Lymphoma

**DOI:** 10.7759/cureus.61593

**Published:** 2024-06-03

**Authors:** Fabian T. H. Ullrich, Nina Hesse, Denis Poddubnyy, Alla Skapenko, Hendrik Schulze-Koops

**Affiliations:** 1 Department of Medicine IV, University Hospital, Ludwig Maximilian University of Munich (LMU) Division of Rheumatology and Clinical Immunology, Munich, DEU; 2 Department of Radiology, University Hospital, Ludwig Maximilian University of Munich (LMU), Munich, DEU; 3 Department of Gastroenterology, Infectious Diseases and Rheumatology, Charité Berlin University Medicine, Berlin, DEU

**Keywords:** axial psoriatic arthritis, psoriatic arthritis, inflammatory back pain, spondyloarthritis, malignant lymphoma, follicular lymphoma, psoriasis vulgaris

## Abstract

Inflammatory back pain is a characteristic of spondyloarthritis. It is not, however, an exclusive symptom of inflammatory rheumatic diseases as it can also be associated with non-inflammatory entities. Infrequently, the etiology can be found in neoplastic conditions such as malignant lymphoma. Even in the presence of comorbidities indicatory of underlying rheumatic disease, like psoriasis vulgaris, the clinician should not be led astray. It is essential to pay attention to contradictory findings, as treatment crucially differs depending on diagnosis. Herein, we report on a psoriasis patient who presented with characteristic inflammatory back pain and deceptive imaging results. While the patient was initially thought to suffer from an inflammatory rheumatic disease with axial involvement, it was the accompanying atypical circumstances, particularly her age, that instantly challenged the diagnosis of axial psoriatic arthritis. She was eventually diagnosed with stage IV follicular lymphoma that manifested with rare and exclusively extranodal lesions and spondyloarthritis-like morphology. This case effectively demonstrates the importance of a thorough diagnostic workup and how certain clinical factors, such as the patient’s age, should be considered when confronted with inflammatory back pain.

## Introduction

In the course of the disease, around 30% of patients with psoriasis vulgaris develop joint involvement in the form of psoriatic arthritis (PsA). Axial involvement manifests in approximately 25-70% of PsA cases [1]. Diagnosing axial PsA (axPsA) or other entities of spondyloarthritides (SpA) based solely on imaging is difficult. A correct diagnosis most often requires a combination of medical history, clinical findings, laboratory results, and different imaging modalities [2]. One clinical characteristic of axial inflammation in spondyloarthritis, including axPsA, is inflammatory back pain. In contrast to degenerative back pain, this symptom is defined by its first occurrence before the age of 40-45 years, morning stiffness, and a nocturnal peak with awakening. Pain intensity is further dependent on levels of physical activity and treatment, which have the potential to either aggravate or alleviate the symptoms [3]. Inflammatory back pain typically presents with an insidious rather than acute onset and should not be classified as such if it is self-limiting within three months [3,4]. A feature of axial involvement in PsA is asymmetric sacroiliitis and involvement of the cervical spine [1].

However, rare and primarily non-inflammatory comorbidities that manifest independently of psoriasis can also mimic the characteristics of axial inflammation. This may result in a potentially fatal delay in diagnosis. It is therefore crucial to constantly scrutinize seemingly unambiguous diagnoses by reevaluating clinical and imaging findings.

## Case presentation

Medical history

The patient, a 49-year-old female with psoriasis vulgaris with nail and scalp involvement since the age of four, presented with chronic back pain under suspicion of axial PsA/axial SpA (axSpA). Pain in the lumbar and sacroiliac joint (SIJ) region had typical inflammatory characteristics (improvement by movement and non-steroidal anti-inflammatory drugs, worsening with rest and physical inactivity, nocturnal peak with awakening), had been present for five months, and was progressive [3]. She could not recall trauma or a history of back pain before the current episode. There were no peripheral musculoskeletal symptoms including joint pain or swelling, signs of enthesitis (e.g., heel pain), or signs of tendinitis (e.g., dactylitis). The only pre-existing medical conditions or procedures she reported were orthopedic surgery of the right cruciate ligament following a skiing accident 11 years ago and surgery of the nasal septum 16 years ago. She denied gastrointestinal symptoms indicative of inflammatory bowel disease and any type of ocular symptoms indicative of uveitis.

Diagnostic findings

**Figure 1:**
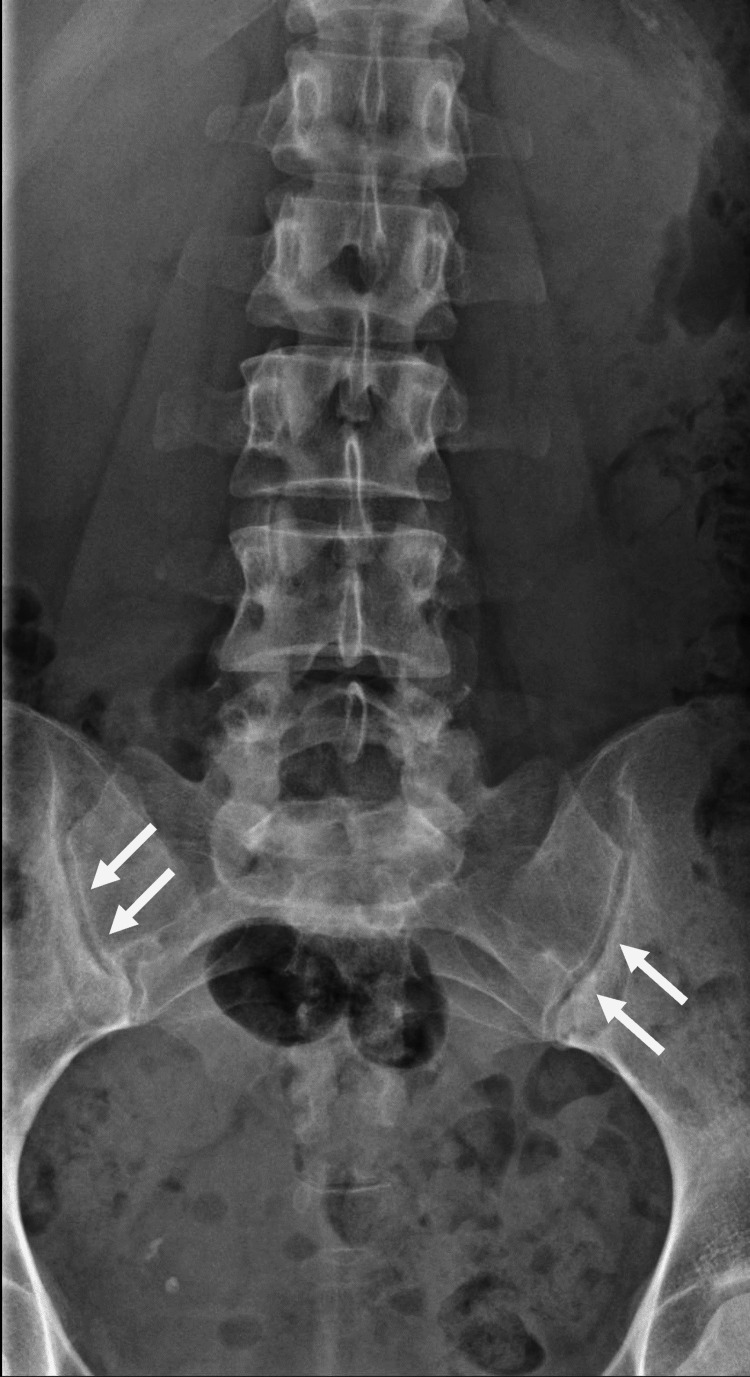
Conventional X-ray Bilateral subchondral sclerosis (arrows) of the sacroiliac joints with discrete erosive changes in posterior-anterior projection. No apparent abnormalities in the lumbar spine.

Besides psoriatic nail discoloration, physical examination was unremarkable. Laboratory results were normal and human leucocyte antigen (HLA)-B27 was negative. Plain radiographs of the SIJ showed discrete bilateral erosive damage and subchondral sclerosis consistent with low-grade SpA (modified New York criteria, radiological grade I-II, arrows in Figure 1) [5]. 

**Figure 2:**
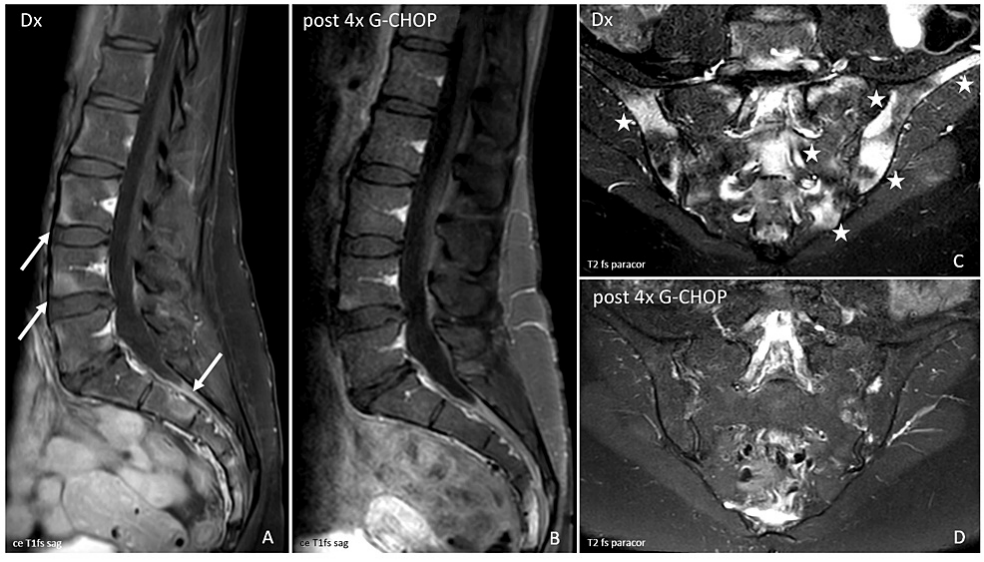
Magnetic resonance imaging Contrast-enhanced T1 fat-saturated (fs)-sequence with sagittal slides (ce T1 fs sag) of the lower thoracic spine, lumbar spine, and sacral bone (A, B) and T2 fs-sequence with paracoronal slides (T2 fs paracor) of the sacroiliac joints (SIJ) (C, D). Intense contrast uptake of lesions in the area of the entheses of the vertebral body endplates and the sacral bone (arrows in A). Bone marrow edema is represented by T2-hyperintense lesions in the medullary cavity of the pelvis (massae laterales) in contact with the articular surfaces of the SIJ and remote from the joint in the os ilium (stars in C). Partial remission of the lesions six months later after four cycles of G-CHOP (post 4x G-CHOP) (B, D). Dx = imaging at first diagnosis; G-CHOP = obinutuzumab plus cyclophosphamide, doxorubicin, vincristine, and prednisone.

Additionally, atypical lesions were apparent in extended imaging workup. Magnetic resonance imaging (MRI) showed profuse contrast uptake of these lesions in the massae laterales adjacent to the SIJ, the pelvic skeleton, and the endplates of the lumbar and lower thoracic spine (Figure 2, arrows in A, contrast-enhanced T1 fat-saturated (fs)-sequence, sagittal slide) with bone marrow edema (Figure 2, stars in C, T2 fs-sequence, paracoronal slide), appearing like spondylitis marginalis. 

Computed tomography-guided biopsy of the os ilium and subsequent staging revealed extra-lymphatic CD20-positive(+)/CD10+/CD79a+/CD23+ follicular lymphoma, World Health Organization (WHO) grade I/II, stage IVB without lymphadenopathy.

Therapy and outcome

Following diagnosis, systemic immunochemotherapy with the anti-CD20-antibody obinutuzumab, cyclophosphamide, doxorubicin, vincristine, and prednisone (Obi/G-CHOP) was initiated. This resulted in a rapid improvement in symptoms. Six months later after four cycles of immunochemotherapy, MRI exhibited partial remission of the lesions (Figures 2B-2D). Final imaging after six therapy cycles during the maintenance phase indicated complete functional remission. At one-year follow-up, the patient was completely pain-free.

## Discussion

Back pain is a well-known symptom of skeletal malignancies, but a typical inflammatory character is rare and has not been described in primary extranodal follicular lymphoma [6]. The group of SpA, however, is clinically characterized by inflammatory back pain, although with a lower prevalence in axPsA than in axSpA [7]. Except for the age criterion (i.e., younger than 40-45 years), our patient presented with the typical clinical characteristics of inflammatory back pain [3]. She further exhibited MRI lesions consistent with the diagnosis of axPsA. Bone marrow edema and inflammation at the insertions of ligaments and joint capsules (entheses), particularly at the anterior and posterior corners of the vertebral endplates are characteristic signs of spondyloarthritis [8].

In our case, by imitating a SpA-like morphology, the location of the neoplastic lesions represents a diagnostic challenge. The magnetic resonance image convincingly illustrates the misleading localization of the lymphomatous lesions close to the entheses and SIJ and hereby provides a reasonable explanation for the inflammatory pain characteristics. Therefore, the simultaneous occurrence of inflammatory back pain and psoriasis must not automatically result in the diagnosis of axPsA. Despite suitable comorbidities, life-threatening diseases should be ruled out before the cause of inflammatory back pain is labeled primarily inflammatory. Atypical circumstances can assist in finding the right diagnosis. As the onset of axial disease in patients older than 45 years is considered exceptional, the patient’s unusually high age for the first manifestation of SpA is of diagnostic importance [9]. Additionally, lesions remote from the joint are indicative of a non-inflammatory origin. Table 1 provides a comprehensive overview of the differential diagnoses of low back pain.

**Table 1:** Differential diagnoses of low back pain Etiologies of low back pain sorted by disease group, specific diagnoses, and indicatory symptoms and findings. The numbers in parentheses indicate the estimated prevalence of the disease group among adult patients, which may vary significantly depending on healthcare providers and local demographics [4,10].

Disease group	Diagnosis	Symptoms and findings
Non-specific low back pain (70%)	Variable (“lumbar sprain/strain”, “idiopathic back pain”)	No specific findings or pathoanatomical confirmation (imaging, laboratory); spontaneous resolution within weeks after onset
Orthopedic disease and trauma (25%)	Degenerative disk and facet-joint disorders	Higher age, degenerative pain character
	Spinal canal stenosis	Claudicatio intermittens spinalis
	Disk herniation, spondylolisthesis	(Pseudo-)radicular pain, neurological symptoms
	Fractures (traumatic, osteoporotic)	History of trauma/osteoporosis
Neoplastic disease (1%)	Lymphoma (multiple myeloma, Hodgkin- and non-Hodgkin-lymphoma) and leukemia	History of cancer, unexplained weight loss, fever, night sweat, anemia, hypercalcemia
	Metastatic cancer (e.g., pulmonary, prostate, breast)	
	Primary cord and bone tumors	
	Retroperitoneal tumors	
Primary inflammatory-rheumatic disease (<1%)	Axial spondyloarthritis	Inflammatory pain character, human leucocyte antigen (HLA)-B27 association, comorbidities (psoriasis vulgaris, inflammatory bowel disease, uveitis, previous infections), enthesitis
	Axial psoriatic arthritis	
	Enteropathic arthritis	
	Reactive arthritis	
	Undifferentiated spondyloarthritis	
Infectious disease (<1%)	Spondylodiscitis/osteomyelitis	Fever, intravenous drug use, chronic/pre-existing infections (e.g., endocarditis, tuberculosis)
	Epidural/(para)spinal abscess	
	Bone tuberculosis	
Visceral disease (2%)	Abdominal aortic aneurysm	Palpable abdominal mass, hypotonia, and shock with rupture
	Urogenital: urolithiasis, pyelonephritis, ovarian torsion, endometriosis	Nausea, colicky pain, dysuria
	Acute pancreatitis	“Belt-like pain”, lipasemia, nausea
	Psoas muscle abscess	Movement-induced pain, risk factors (e.g., appendicitis)

The progression of the patient's complaints over several months along with its specific and reproducible pain character were clinical evidence that contradicted the presence of non-specific back pain [4]. Once the imaging results were available, orthopedic disease and trauma could be ruled out. The laboratory results and the patient's medical history did not indicate an infection or visceral disease [10].

Findings that are associated with axPsA can further be helpful in differentiating axial inflammatory disease from other causes of back pain. Concomitant peripheral arthritis, HLA-B27 positivity, asymmetric sacroiliitis, and cervical involvement are frequently found in axPsA and were absent in our patient [1]. The rapid clinical response to an immunochemotherapy regimen that would not be expected to resolve symptoms in an axial rheumatic disease and the associated complete resolution of the axial lesions support the causality between the extranodal follicular lymphoma and the reported symptoms.

## Conclusions

In conclusion, inflammatory back pain, although usually regarded as a domain of inflammatory rheumatic diseases, can also be indicative of more life-threatening conditions like malignant lymphoma. The patient presented here is a remarkable illustration of how useful established clinical characteristics of inflammatory rheumatic diseases can be when making a diagnosis. Our case highlights that coincidence needs to be differentiated from causality by appreciating deceptive imaging results and applying a profound clinical assessment. To the best of our knowledge, this is the first report of a stage IV follicular lymphoma that manifested with rare and exclusively extranodal lesions and a spondyloarthritis-like morphology causing inflammatory back pain.
